# Supplementary material for: Association between oral microbiome and seven types of cancers in East Asian population: a two-sample Mendelian randomization analysis
Source: Front Mol Biosci. 2023 Nov 21;10:1327893. doi: 10.3389/fmolb.2023.1327893 (PMC10702768; doi:10.3389/fmolb.2023.1327893)
Supplement: Supplementary file 1 [file DataSheet1.ZIP › Supplementary Materials/MR plots for tongue/tongue═╝/Ovarian cancer/pheno.3188_to_ovarian cancer_leave_one_out.pdf]

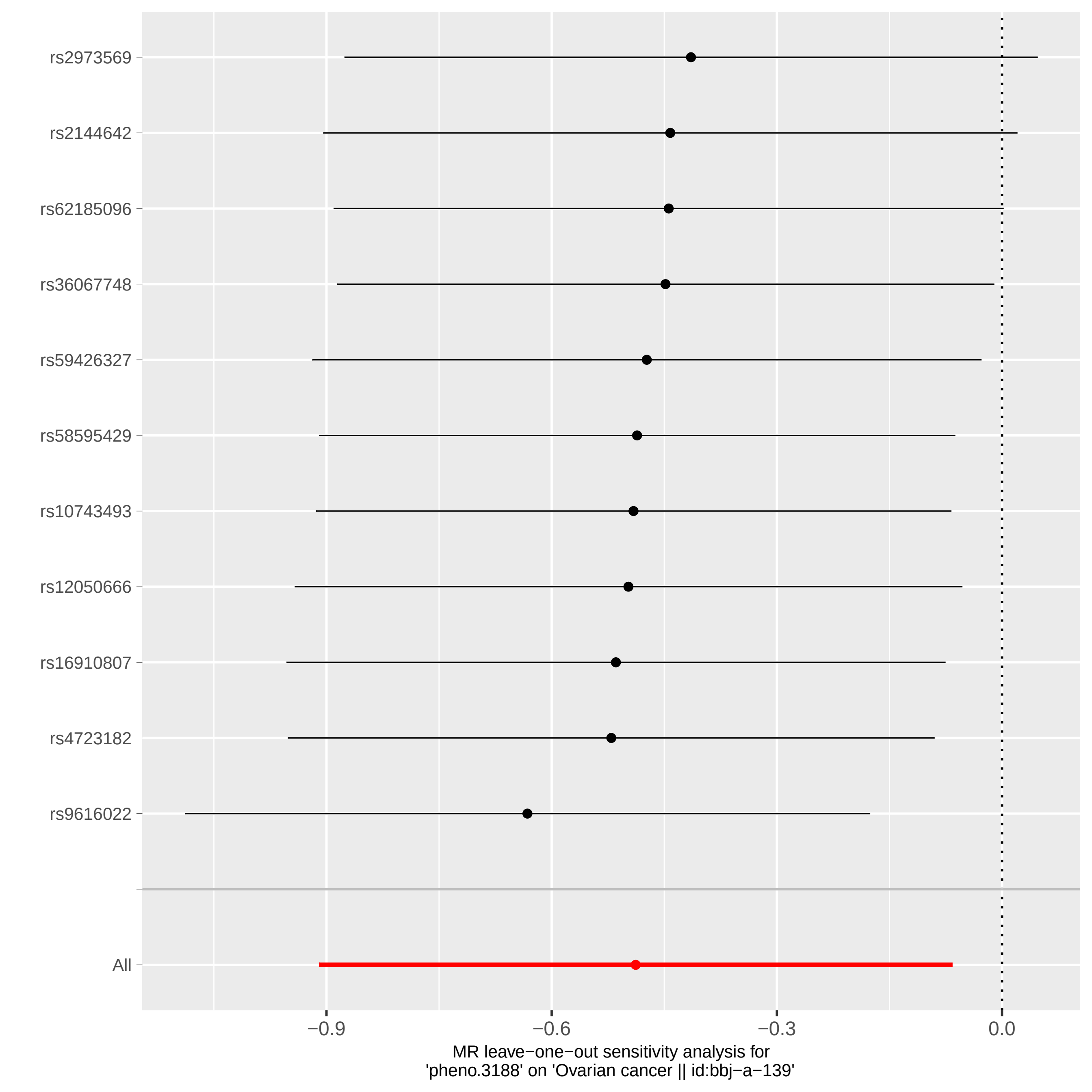
rs2973569
rs2144642
rs62185096
rs36067748
rs59426327
rs58595429
rs10743493
rs12050666
rs16910807
rs4723182
rs9616022
All
−0.9
−0.6
−0.3
0.0
MR leave−one−out sensitivity analysis for
'pheno.3188' on 'Ovarian cancer || id:bbj−a−139'
